# Polypharmacy is a risk factor for hospital admission due to a fall: evidence from the English Longitudinal Study of Ageing

**DOI:** 10.1186/s12889-020-09920-x

**Published:** 2020-11-26

**Authors:** P. Zaninotto, Y. T. Huang, G. Di Gessa, J. Abell, C. Lassale, A. Steptoe

**Affiliations:** 1 Department of Epidemiology and Public Health, University College London, 1-19 Torrington Place, London, WC1E 7HB UK; 2 Department of Behavioral Science and Health, University College London, London, UK; 3 Programme of Epidemiology and Public Health, Hospital del Mar Medical Research Institute (IMIM), 08003 Barcelona, Spain

**Keywords:** Older people, Polypharmacy, Falls, Hospitalization

## Abstract

**Background:**

Falls amongst older people are common; however, around 40% of falls could be preventable. Medications are known to increase the risk of falls in older adults. The debate about reducing the number of prescribed medications remains controversial, and more evidence is needed to understand the relationship between polypharmacy and fall-related hospital admissions. We examined the effect of polypharmacy on hospitalization due to a fall, using a large nationally representative sample of older adults.

**Methods:**

Data from the English Longitudinal Study of Ageing (ELSA) were used. We included 6220 participants aged 50+ with valid data collected between 2012 and 2018.The main outcome measure was hospital admission due to a fall. Polypharmacy -the number of long-term prescription drugs- was the main exposure coded as: no medications, 1–4 medications, 5–9 medications (polypharmacy) and 10+ medications (heightened polypharmacy). Competing-risk regression analysis was used (with death as a potential competing risk), adjusted for common confounders, including multi-morbidity and fall risk-increasing drugs.

**Results:**

The prevalence of people admitted to hospital due to a fall increased according to the number of medications taken, from 1.5% of falls for people reporting no medications, to 4.7% of falls among those taking 1–4 medications, 7.9% of falls among those with polypharmacy and 14.8% among those reporting heightened polypharmacy**.** Fully adjusted SHRs for hospitalization due to a fall among people who reported taking 1–4 medications, polypharmacy and heightened polypharmacy were 1.79 (1.18; 2.71), 1.75 (1.04; 2.95), and 3.19 (1.61; 6.32) respectively, compared with people who were not taking medications.

**Conclusions:**

The risk of hospitalization due to a fall increased with polypharmacy. It is suggested that prescriptions in older people should be revised on a regular basis, and that the number of medications prescribed be kept to a minimum, in order to reduce the risk of fall-related hospital admissions.

## Background

Falls, defined as an unanticipated incident in which a person come to rest on the ground or a lower level, [1] are the most frequent type of accidents among older people [2]. One in three people over 65 years of age experience at least one fall each year, and injuries occur in approximately 20% of such cases [3]. Older people who have suffered a fall experience an increased risk of recurrence and of being hospitalized. Falls not only carry a human burden, but they can incur considerable medical care costs, with estimates being suggested between 0.85 and 1.5% of total healthcare expenditure in the UK [4].

It has been estimated that around 40% of falls in older people are preventable [5]. As a consequence, a large body of research has emerged to explore risk factors that might determine whether someone is at risk of experiencing a fall, especially a fall for which they might require treatment in hospital. Polypharmacy, defined as the chronic co-prescription of multiple medications, has been identified as one of the most significant factors associated with falls among older people [6, 7]. Several studies in ageing populations have reported that the risk of a fall increases with the use of four or more medications [8–16]. However, older adults using multiple medications might also have several long-term conditions, whose pharmacological treatment often requires the concomitant use of several medications. Therefore, the risk of falls might not be independent of these long-term conditions. Indeed, those with multimorbidity (defined as reporting three or more long-term conditions) who also take multiple medications have a higher risk of falls [17].

Recent studies also suggest that medications such as cardiovascular agents, central nervous system drugs, analgesics and endocrine drugs, increase the risk of falls [14, 15]. The possible underlying mechanisms for the increased risk of falls related to the use of these medications, called “fall risk-increasing drugs” (FRIDs), relate to the adverse effects (eg, dizziness, imbalance or mobility difficulties, reduced attention and vigilance). However, Seppala et al., in their systematic review, point out that adjustment for long-term conditions and “fall risk-increasing drugs” has rarely been carried out in studies of polypharmacy and falls [18]. Properly adjusting for both is imperative, since polypharmacy is often the consequence of long term conditions, [19] and the risk of polypharmacy on falls may not be independent of “fall risk-increasing drugs” [14].

The debate about reducing the number of prescribed medications remains controversial. On the one hand, the prescription of several medications is largely justified by the complex clinical profile of older adults and it has been shown that interventions to reduce the number of concurrent medications have been unsuccessful [20–22]. On the other hand, studies have shown that medication withdrawal, especially FRIDs, has been effective in reducing the risk of falls [23]. Therefore, more evidence is needed to understand the relationship between polypharmacy and fall-related hospital admissions. Large nationally representative longitudinal studies of ageing, which collect a broad range of factors and have been linked to administrative health data, are best placed to provide insights into this relationship. Accordingly, the aim of this study is to examine, in a nationally representative sample of older adults, whether polypharmacy is a risk factor for hospitalization due to a fall. We will examine this independently of other risk factors, including long-term conditions and drugs known to increase the risk of falling (FRIDs).

## Methods

### Data

These data are from the English Longitudinal Study of Ageing (ELSA) [24] a nationally representative sample of individuals aged 50 and older living in private households in England, followed and re-interviewed every 2 years. The main objective of the study is to understand the complex dynamics of the ageing process, that is the relationships between economic and family circumstances, behaviour, social participation, biology, retirement, and health and well-being. The study began in 2002–2003 (first phase of data collection referred to as wave 1). Data collection comprises face-to-face interviews, self-completion questionnaires and nurse visits in participants’ homes every other wave. For the purpose of this study, we used data from wave 6 (2012–2013) as our baseline, when information on medication was first collected during the nurse visit. A total of 6220 individuals had valid data on medications and covariates of interest at baseline. All individuals included in the baseline sample (2012–2013) had their data linked to Hospital Episode Statistics, and to mortality even those who dropped out of the study after baseline.

#### Outcome measure

Hospitalization due to a fall was derived from the Hospital Episode Statistics data linked by NHS digital to ELSA participants’ NHS number, date of birth, gender and postcode. All participants were followed-up from the interview date up to March 2018. For each participant, a record of each hospitalization to secondary care is available, with admission date, episode duration, primary diagnosis and secondary diagnoses. Diagnoses are coded according to the international classification of disease 10th version (ICD-10). Falls correspond to the ICD-10 codes W00 to W19. The event “fall” is defined as the first episode where a primary diagnosis of fall was recorded.

#### Exposure: polypharmacy

At wave 6 (2012–2013) during the nurse visits to participants’ homes nurses recorded medications taken by each participant. These drugs, both generic and brand name, were allocated codes based on the British National Formulary. In the definition of polypharmacy, only long-term medications were considered. Long-term medications were either drugs for long-term diseases such as diabetes and hypertension, or drugs for long-term symptoms such as sedatives. Despite variation, which exists in the definition of polypharmacy, the number of medications in this sample was recoded according to the most commonly used cut-offs: No medications, 1–4 medications, 5–9 medications (polypharmacy) and 10 or more medications (heightened polypharmacy) [25].

#### Potential confounders

Socio-demographic variables included age (continuous, ranging from 54 to 101 at wave 6), sex (males vs females), cohabitation status (currently living or not with a partner whether married or not), and educational attainment (high-college and above, medium-A-levels, low-below O-levels). For cognitive function we used a memory score computed from a word-list learning test [26] in which a list of ten words was read out to study participants, who were then asked to recall as many words as possible immediately and after around a five-minute delay (total score ranged from 0 to 20 with higher scores indicating better cognitive function). Health behaviours included frequency of alcohol intake (in days) in the last 12 months ascertained from self-reported responses and coded as daily (5/7 days week) or less than daily (< 5 days a week); smoking status (non-smoker vs current smoker); body mass index (computed from objectively measured height and weight); physical activity (active vs sedentary). Physical activity was measured using responses to questions on leisure-time physical activity and aggregated to compute a five-level score from inactive to active, and used in the analysis as binary. We also used a self-reported measure of eyesight (poor vs good). Health conditions were ascertained from self-reported doctor diagnosis and included: coronary heart disease (CHD), stroke, diabetes, depression (defined as 4 or more depressive symptoms), respiratory illness, arthritis, cancer, dementia, Parkinson’s disease and Alzheimer’s disease. In addition we computed a variable for multimorbidity by recoding the number of long-term conditions reported into a dichotomous variable, with a cut-off of 3 or more [16]. FRIDs were also taken into account as a binary variable (2+ FRIDS versus none). FRIDs included cardiovascular agents, central nervous system drugs (not including antiparkinsonians), analgesics (non-steroidal anti-inflammatory drugs), thyroid drugs, and antihyperglycemic drugs [14]. Physical functioning was measured using number of limitations with mobility items (continuous) and the number of difficulties with activities of daily living (ADLs) and instrumental activities of daily living (IADLs) (binary, one or more versus no difficulties), and cognitive function. ADLs items were: dressing, walking across a room, bathing or showering, eating, getting out of bed, using the toilet; IADLs items were: using a map, preparing a hot meal, shopping for groceries, making phone calls, taking medications, doing work around the house, managing money.

### Statistical analysis

To examine the association between polypharmacy and hospitalization due to a fall we employed competing-risk regression analysis with subdistribution hazard ratios (SHR) and related 95% Confidence Intervals, using a version of the Fine and Gray method [27]. This method allows a competing risk – an event that might occur during the follow-up instead of the event of interest – to be taken into account in the analysis. In this case, death is a potential competing risk when examining incidence rates of admission to hospital due to a fall; therefore, it is important to take this into account, rather than treating those who had died as censored. Mortality status was ascertained from linked register data, up to the end of March 2018. By the end of this follow-up period (six years) 295 admissions to hospital due to a fall were recorded and 594 deaths occurred.

In a sensitivity analysis, we explored whether fall hospitalization was associated with the use of polypharmacy also among people in the 0–1 FRID category, as previous studies suggested that the risk of polypharmacy of falls might not be associated with fall risk, independently of FRIDs [9, 14].

## Results

The baseline characteristics of the sample in 2012–2013 according to polypharmacy status are reported in Table 1. The prevalence of people admitted to hospital due to a fall increased steadily according to polypharmacy status. This ranged from 1.5% in people reporting no medications, to 4.7% of falls among people reporting 1–4 medications, 7.9% of falls occurred among people with polypharmacy (5–9 medications) and 14.8% among those reporting heightened polypharmacy (10 + medications). Respondents reporting polypharmacy and heightened polypharmacy were also older and reported poorer health outcomes at baseline than those not taking medications.

**Table 1:** Baseline characteristics of participants according to polypharmacy, England 2012–2013

	Polypharmacy				
	No medications	1–4 medications	5–9 medications (polypharmacy)	10+ medications (heightened polypharmacy)	*P* Value
Number of Respondents	1720	3051	1290	159	
Age, years: mean (s.e.)	64.07 (0.23)	66.04 (0.24)	71.00 (0.39)	71.02 (0.81)	< 0.001
Women, % (n)	47.3 (909)	54.5 (1729)	52.3 (675)	50.2 (85)	< 0.001
Hospitalization due to falls by 2018, % (n)	1.5 (33)	4.7 (153)	7.9 (99)	14.8 (25)	< 0.001
Deaths [by March 2018], % (n)	3.1 (63)	8.1 (245)	19.3 (240)	30.8 (46)	< 0.001
Living alone, % (n)	23.5 (430)	28.8 (917)	36.1 (469)	38.7 (66)	< 0.001
Highest level of education, % (n)					
Degree,	22.6 (404)	15.7 (533)	11.5 (148)	11.5 (21)	< 0.001
Intermediate	61.5 (1064)	58.7 (1805)	46.8 (722)	46.8 (81)	
No qualification	15.9 (252)	25.6 (713)	41.7 (420)	41.7 (57)	
Poor vision, % (n)	7.8 (123)	11.1 (314)	21.2 (255)	29.8 (48)	< 0.001
Diagnoses and Health conditions, % (n)					
CHD	0.4 (10)	4.9 (158)	28.5 (355)	53.3 (82)	< 0.001
Diabetes	0.7 (15)	8.2 (235)	30.9 (383)	47.3 (77)	< 0.001
Depression	3.9 (66)	8.1 (224)	11.4 (115)	18.9 (29)	< 0.001
Asthma or lung disease	4.7 (74)	14.6 (437)	25.2 (310)	53.4 (80)	< 0.001
Stroke	0.3 (6)	2.9 (92)	11.2 (142)	17.0 (23)	< 0.001
Cancer	3.2 (59)	5.4 (171)	7.8 (108)	7.3 (13)	< 0.001
Arthritis	20.0 (411)	39.1 (1268)	58.8 (753)	72.0 (117)	< 0.001
Parkinson’s disease	0.0 (1)	0.6 (20)	1.0 (14)	2.6 (4)	< 0.001
Alzheimer’s disease	0.0 (0)	0.1 (3)	0.4 (4)	0 (0)	0.055
Dementia	0.1 (3)	0.7 (18)	1.9 (20)	2.1 (2)	0.001
Has 3+ long-term conditions, % (n)	0.6 (12)	3.2 (100)	22.0 (255)	53.5 (84)	< 0.001
Takes 2+ FRIDs^a^, % (n)	0	30.1 (924)	85.6 (1100)	95.3 (151)	< 0.001
Difficulty in ADL or IADL, % (n)	8.6 (149)	22.1 (649)	49.6 (582)	68.9 (109)	< 0.001
Limitations in mobility, mean (s.e.)	0.69 (0.04)	1.70 (0.05)	3.58 (0.10)	5.57 (0.26)	< 0.001
Hardly ever engage in physical activity, % (n)	5.3 (83)	14.1 (388)	34.8 (381)	55.4 (83)	< 0.001
BMI value: mean (s.e.)	27.19 (0.14)	28.55 (0.11)	30.37 (0.19)	31.57 (0.55)	< 0.001
Current smoker, % (n)	15.0 (205)	12.4 (302)	13.4 (151)	21.7 (32)	0.021
Almost daily alcohol consumption, % (n)	19.7 (382)	20.5 (673)	15.9 (227)	13.7 (22)	0.007
Memory index: mean (s.e.)	11.80 (0.09)	10.92 (0.07)	9.51 (0.11)	8.40 (0.32)	< 0.001

Source: ELSA, Wave 6. Weighted data. ^a^ *FRID* Fall-risk increasing drugs

**Fig. 1:**
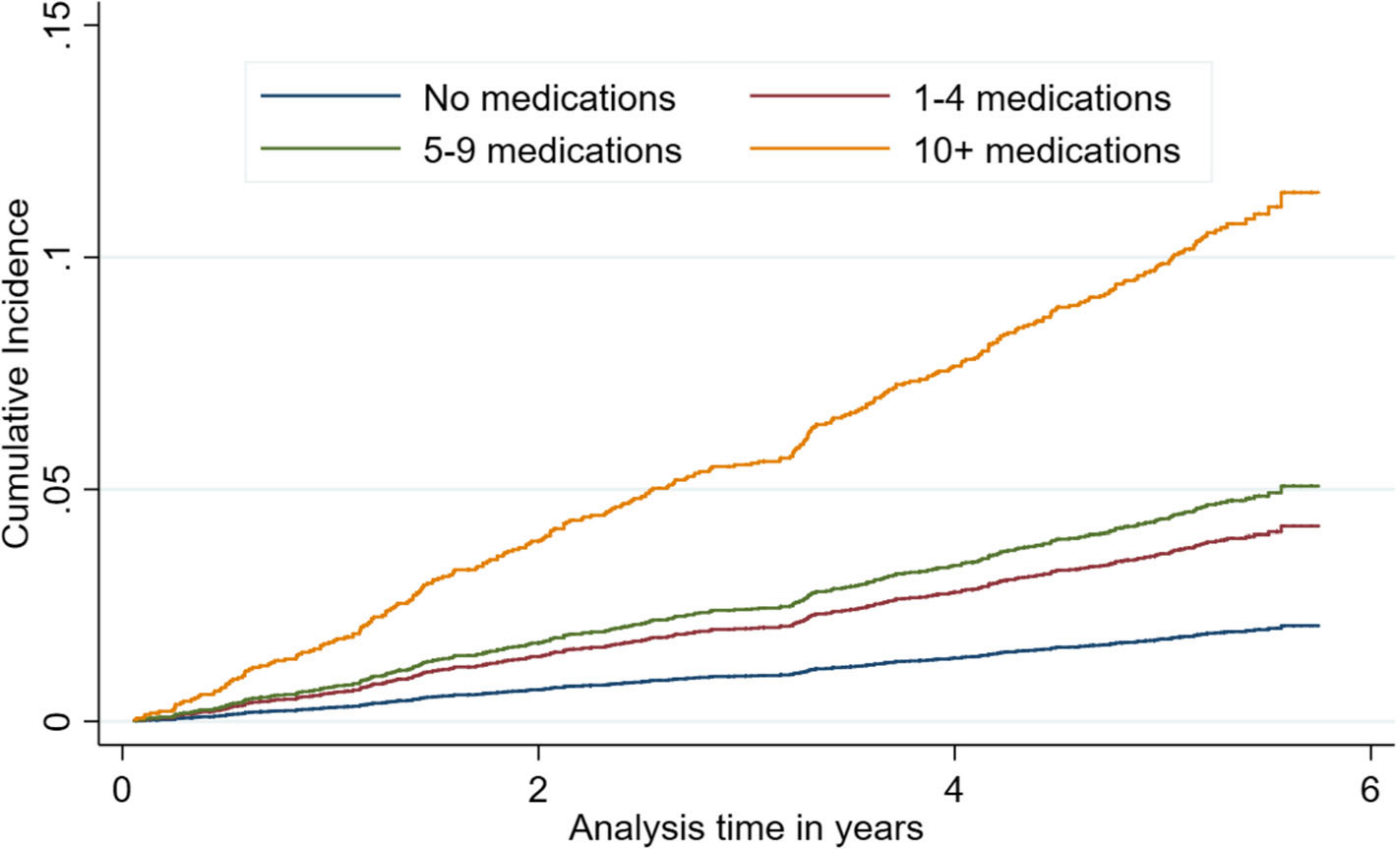
Estimates of the cumulative incidence curves of risk of hospitalization following a fall according to polypharmacy, England 2012–2018

The unadjusted cumulative incidence function shows a dose-response association in the risk of hospitalization due to a fall and polypharmacy; in particular, the cumulative incidence curve for 10+ medications increased steeply with time (Fig. 1).

In Table 2 we report the subdistribution hazard ratios (SHR) for the association between polypharmacy and risk of hospitalization due to a fall estimated using competing risk analysis. The age- and sex- adjusted SHRs for hospitalization due to a fall among people who reported taking 1–4 medications, polypharmacy and heightened polypharmacy were 2.06 (95%CI:1.38;3.07), 2.49 (95%CI:1.62;3.82), and 5.79 (95%CI:3.33;10.1) respectively, compared with people who were not taking medications. After adjustment for all covariates, the association between polypharmacy and hospitalization due to a fall, reduced to 1.79 (95%CI:1.18; 2.71) among people who reported taking 1–4 medications, reduced to 1.75 (95%CI: 1.04; 2.95) among those reporting polypharmacy and was 3.19 (95%CI: 1.61; 6.32) for heightened polypharmacy.

**Table 2:** Subdistribution hazard ratios (95 CIs) for the association between the number polypharmacy and hospitalization following a fall (*N* = 6220), England 2012–2018

	Age and gender adjusted		Fully adjusted^a^	
Polypharmacy	SHR (95%CI)	***P*** Value	SHR (95%CI)	***P*** Value
No medications	1 (Ref)		1 (Ref)	
1–4 medications	2.06 (1.38; 3.07)	< 0.0001	1.79 (1.18; 2.71)	< 0.01
5–9 medications (polypharmacy)	2.49 (1.62; 3.82)	< 0.0001	1.75 (1.04; 2.95)	< 0.05
10+ medications (heightened polypharmacy)	5.79 (3.33; 10.1)	< 0.0001	3.19 (1.61; 6.32)	< 0.001

Hospitalization following a fall *N* = 295, competing event deaths *N* = 594. ^a^Adjusted for age, gender, living alone, education, poor vision, all diagnoses and health conditions, 3+ long-term conditions, 2+ FRIDs, any functional impairment, health behaviours, tests of cognitive function

In further sensitivity analysis, we investigated whether the association between polypharmacy and admissions to hospital due to a fall remained when FRIDs were included in the model. We ran the analyses again among those who were taking 0–1 FRIDs. We found that the association between polypharmacy and hospitalization due to a fall reduced in magnitude, but was still statistically significant by polypharmacy status, including the group of 1–4 medications (SHR 1.65 95%1.1;2.5 *p* = 0.025 compared to not taking medications).

## Discussion

Using a large nationally representative sample of older people in England, our study showed a strong association between polypharmacy status and the risk of hospitalization due to a fall. We found that the risk was highest among people reporting polypharmacy and heightened polypharmacy compared to those who reported taking no medications. We also observed a slightly elevated risk among older adults who reported the concurrent use of 1–4 medications compared with those who reported taking none. These associations were not explained by common risk factors for falls, neither by multi-morbidities nor by FRIDs.

In agreement with results from a population-based case control study of people 65 years and older living in Stockholm, [12] we found that the use of one or more medications led to an increased risk of hospitalization due to a fall. In a previous investigation using data from ELSA [8] it was found that polypharmacy and heightened polypharmacy significantly increased the risk of falls among people aged 60 and over, however, the study only used a short follow-up period (2 years) and a self-reported measure of falls. Our analysis has improved the results of previous studies by using an objective measure of falls and by studying a younger cohort at the first assessment (aged 50 years old and over) [8, 9, 11–14]. Furthermore, we showed that this increased risk of falls among people taking medications was independent of long-term conditions and FRIDs.

Falls are common among older adults, and as the proportion of elderly people in the population continues to increase, falls in this group are predicted to pose a serious burden on healthcare expenditure. Strategies to prevent falls include the identification of potential modifiable risk factors, such as multiple medications [6]. Our results contribute to current discussions in the UK about reducing the number of prescribed medications in older age. The National Institute for Health and Care Excellence published new guidelines for the management of multimorbidity among individuals taking 10 or more prescribed medications. However, we have shown that the risk of hospitalization due to a fall is also high in patients taking 1–4 and 5–9 medications, and these people might be excluded from the target group for medication reviews [28]. Period reviews of prescriptions among older patients should be in place to assure that the number of medications consumed is minimalized, especially amongst frail people who might be at higher risk of falling.

### Strengths and limitations

This study examined the association between polypharmacy and hospitalization due to a fall in a nationally representative sample of non-institutionalised individuals in England. The use of medication data collected by a nurse, and hospital administrative data, is less susceptible to recall bias. Moreover, we considered the number of long-term conditions from which participants suffered and were able to adjust for these in our analyses. Lastly, we used a competing risk analysis strategy to consider mortality as a competing event.

The main limitation of our study is that the information on medication was collected for the first time in 2012/2013; it would have been preferable to have multiple time points of medication records to establish whether the duration of polypharmacy had an impact on the risk of being admitted to hospital due to a fall. We were able to investigate the number of medications prescribed, but unfortunately, we were not able to explore the nature of these medications, since the specific drug name was often not available. We were also not able to test common drug-drug interactions directly, [29] such as with antihypertensive, diuretics and selective serotonin reuptake inhibitors (SSRIs) [30]. Potentially serious drug-drug interactions have been reported in drugs recommended by clinical guidelines for different long-term conditions, such as cardiovascular diseases, type 2 diabetes, depression and dementia [29, 31]. Severe drug-drug interactions could happen between SSRIs and serotonin–norepinephrine reuptake inhibitors or between beta-blockers and certain antiarrhythmic agents; we cannot entirely exclude that these interactions occurred in our sample [29, 31]. Future studies should examine specific drug-drug interactions in detail [31].

An additional issue is that the assessment of polypharmacy was based on the long-term medications that were being taken by participants at the time of the nurse visits. Although excluded medications were primarily painkillers, a small proportion of antihistamines, both sedating and non-sedating types, were also excluded. Furthermore, we do not have information about whether the medication prescribed changed over the follow-up period. People might also have taken other medications acutely that might have provoked problems with balance and increased risk of falls.

It is also possible that the risk of falls may be increased by strong doses of FRIDs, but we did not have information on medication dosage. Finally, although we used a wide range of confounders, some residual confounding might exist. For example, we could not adjust for objective measures of physical functioning since those were collected only among those aged 60 and over.

## Conclusions

In conclusion, we found that the risk of hospitalization due to a fall increased with polypharmacy status. The increased risk was apparent among those reporting polypharmacy and heightened polypharmacy, but also among those reporting the concurrent use of 1–4 medications. It is advisable that drug prescriptions in older people be revised on a regular basis, and that the number of medications should be kept to the minimum possible as it might reduce the risk of fall-related hospital admissions.

## Data Availability

The ELSA datasets are available in the UK Data Service, [https://beta.ukdataservice.ac.uk/datacatalogue/series/series?id=200011]. Hospital Episode Statistics data and mortality data will be made available in due course.

## Abbreviations

ADLs: Activities of Daily Living
CHD: Coronary Heart Disease
ELSA: English Longitudinal Study of Ageing
FRIDs: Fall Risk-Increasing Drugs
IADLs: Instrumental Activities of Daily Living
ICD-10: International Classification of Disease
SHR: Subdistribution Hazard Ratios
